# Effects of an eHealth Literacy Intervention for Older Adults

**DOI:** 10.2196/jmir.1880

**Published:** 2011-11-03

**Authors:** Bo Xie

**Affiliations:** ^1^University of MarylandCollege of Information StudiesCollege Park, MDUnited States

**Keywords:** Health literacy, lifelong learning, aged, technology

## Abstract

**Background:**

Older adults generally have low health and computer literacies, making it challenging for them to function well in the eHealth era where technology is increasingly being used in health care. Little is known about effective interventions and strategies for improving the eHealth literacy of the older population.

**Objective:**

The objective of this study was to examine the effects of a theory-driven eHealth literacy intervention for older adults.

**Methods:**

The experimental design was a 2 × 2 mixed factorial design with learning method (collaborative; individualistic) as the between-participants variable and time of measurement (pre; post) as the within-participants variable. A total of 146 older adults aged 56–91 (mean 69.99, SD 8.12) participated in this study during February to May 2011. The intervention involved 2 weeks of learning about using the National Institutes of Health’s SeniorHealth.gov website to access reliable health information. The intervention took place at public libraries. Participants were randomly assigned to either experimental condition (collaborative: n = 72; individualistic: n = 74).

**Results:**

Overall, participants’ knowledge, skills, and eHealth literacy efficacy all improved significantly from pre to post intervention (*P* < .001 in all cases; effect sizes were >0.8 with statistical power of 1.00 even at the .01 level in all cases). When controlling for baseline differences, no significant main effect of the learning method was found on computer/Web knowledge, skills, or eHealth literacy efficacy. Thus, collaborative learning did not differ from individualistic learning in affecting the learning outcomes. No significant interaction effect of learning method and time of measurement was found. Group composition based on gender, familiarity with peers, or prior computer experience had no significant main or interaction effect on the learning outcomes. Regardless of the specific learning method used, participants had overwhelmingly positive attitudes toward the intervention and reported positive changes in participation in their own health care as a result of the intervention.

**Conclusions:**

The findings provide strong evidence that the eHealth literacy intervention tested in this study, regardless of the specific learning method used, significantly improved knowledge, skills, and eHealth literacy efficacy from pre to post intervention, was positively perceived by participants, and led to positive changes in their own health care. Collaborative learning did not differ from individualistic learning in affecting the learning outcomes, suggesting the previously widely reported advantages of collaborative over individualistic learning may not be easily applied to the older population in informal settings, though several confounding factors might have contributed to this finding (ie, the largely inexperienced computer user composition of the study sample, potential instructor effect, and ceiling effect). Further research is necessary before a more firm conclusion can be drawn. These findings contribute to the literatures on adult learning, social interdependence theory, and health literacy.

## Introduction

Health literacy is “the degree to which individuals have the capacity to obtain, process, and understand basic health information and services needed to make appropriate health decisions” [1]. This concept has drawn much attention recently [2-6], with increasing well-documented evidence of both the negative impact of poor health literacy on health outcomes and health care costs [7] and the alarmingly low levels of health literacy among American adults: a national survey showed only 12% of the adults in the United States have proficient health literacy, and this proportion drops to 3% among older adults [8].

Recently, information and communication technologies (ICTs) are increasingly being widely used in health care [9,10], presenting both opportunities and challenges for developing and implementing effective health literacy interventions. As government agencies such as the US National Institutes of Health (NIH), nonprofit organizations such as medical associations, and for-profit organizations alike are increasingly putting health information online, the Internet has already become an invaluable resource for high-quality health information [11-13]. This resource, however, can only be useful if the user has adequate eHealth literacy, or “the ability to seek, find, understand, and appraise health information from electronic sources and apply the knowledge gained to addressing or solving a health problem” [14]. Individuals who have low health literacy—for instance, older adults—are likely to also have low computer and Internet literacy [15-17], thus facing a double jeopardy in the eHealth era.

Existing literature provides little scientific evidence about effective health literacy interventions [18], and even less about effective interventions for improving the eHealth literacy of the older population. Existing interventions focus predominantly on simplifying medical materials and instructions [19-21]. This “lowering-the-bar” approach is useful but limited given the complexity of medical terminology and knowledge. Education and training is another key approach to addressing the health illiteracy crisis [22]. This approach requires an understanding of health literacy as an active, lifelong learning process that features continuous learning of new, valid health information and unlearning of outdated, harmful information [22]. Such an understanding is especially important in the context of ICTs being increasingly used by health consumers, professionals, and policy makers alike in health care [9,10]. As ICTs change at a rapid rate, so do the requirements for health literacy skills [23].

### Electronic Health Information for Lifelong Learners

The present study is a part of the Electronic Health Information for Lifelong Learners (*e*HiLL) research project that aims to address these gaps in the literature [4-6,24]. The goal of the larger *e*HiLL research project is to generate scientific knowledge about optimal learning conditions and strategies that can effectively and efficiently improve older adults’ learning and use of eHealth applications. To achieve this goal, the *e*HiLL research project consists of a series of experimental studies designed to examine the effects of various learning conditions and strategies through theory-driven, hypothesis-testing, rigorous experiments. The *e*HiLL experimental studies build on an understanding of health literacy as an active, lifelong learning process that goes beyond the formal educational settings in early life stages [22]. Importantly, guided by the literature on older adults’ learning of computer technology, the *e*HiLL interventions all feature key elements designed specifically to accommodate older computer learners’ needs and preferences [4]. These include (1) providing step-by-step, detailed instructions and avoiding technical jargon [24,25], (2) providing hands-on practice and encouraging questions [26], (3) making sure each lesson builds on previous lessons and increases complexity gradually [25,27], (4) ensuring the learners experience at least some level of success at the initial stage of the training [25,28,29], (5) conducting the training in a familiar, relaxed, and supportive environment [27,29], and (6) offering the training in the early morning hours, which is generally the optimal time of day for older learners [30].

These key elements were fully incorporated in prior *e*HiLL interventions and proven to be effective in improving older adults’ eHealth literacy [4-6,24]. Building on and expanding the success of these prior studies, the present study fully incorporates these key elements while adding a new aspect—that is, collaborative versus individualistic learning. Existing literatures on adult learning and cognitive development in later life provide a theoretical foundation for the collaborative versus individualistic versions of the intervention tested in the present study. The present study differs from prior *e*HiLL studies in important ways. First, the only prior *e*HiLL study [6] that involved both collaborative and individualistic learning had a brief intervention time (the experimental session lasted 2 hours) and it used a 16-minute-long video tutorial as the curriculum. In comparison, the present study involved 2 weeks of intervention for a total of 8 hours, using a paper-based curriculum. Second, while the present study and prior *e*HiLL studies [4,5,24] all used instructional materials drawn from the same tutorial, the Xie and Bugg [4] and Xie [24] studies focused on individualistic learning and the Xie [5] study was only on collaborative learning. The present study is the first *e*HiLL study that compares the relative effects of collaborative versus individualistic learning over an extended period of intervention time.

### Collaborative Versus Individualistic Learning

Collaborative learning is one of the most common forms of active learning. It can be defined as “any instructional method in which students work together in small groups toward a common goal,” ([31] p 223). Collaborative learning requires learners to actively engage in the learning process by engaging in meaningful activities and reflecting on what they are learning from doing those activities [32]. Collaborative learning is often contrasted with individualistic learning that features students working on their own with little or no interaction with peer students [33].

The superiority of collaborative learning over individualistic learning is predicted by social interdependence theory, which emphasizes the interdependence among group members by arguing that the group is a “dynamic whole,” such that any change in the state of a group member changes that of other group members [33]. The social interdependence among group members can be positive, negative, or nonexistent. Positive interdependence (collaboration) facilitates learning by promoting collaboration among group members. It can be found when individuals recognize that the only way they can achieve their goals is when other group member also achieve their goals. Negative interdependence (competition) exists when individuals recognize that the only way they can achieve their goals is when others fail to do so. It often results in obstructive interactions impeding learning. Nonexistent interdependence (individualistic efforts) exists when individuals perceive that their achievement is not affected by others’ performance. It features no interaction among group members as each member learns independently [33,34].

Extensive empirical evidence supports the effectiveness of collaborative learning. A meta-analysis of over 300 studies shows that collaborative learning outperforms individualistic and competitive learning in postsecondary and professional settings [34]. However, there are still major gaps in the literature that require further examination. Collaborative learning research within the social interdependence tradition is predominantly based on formal education of younger adults [33,34]. Whether these findings can be generalized to older age groups in informal educational settings is yet to be answered.

Recently, the cognitive-developmental literature has begun to examine collaborative learning as a mechanism for improving cognitive abilities in later life [35-37]. Some (non-computer-related) studies find a positive impact of collaborative learning on older adults’ performance [38,39]. However, there is also evidence that, compared with individualistic learning, collaborative learning has no, or even negative, impact [40-42]. A possible reason might be that existing research within the cognitive-developmental tradition generally does not provide detailed instructions to ensure collaboration [42]. Instead, participants are simply instructed to “work together” [43] or “collaborate as much as possible” [40]. To ensure collaborative learning for older adults in informal settings, it is critical to develop effective strategies that really work.

### Group Composition

Group composition may affect the “dynamic whole” of a group and, subsequently, the learning process and outcomes [33,34]. Evidence suggests there is more collaboration in groups with either female- or male-gender majority than in groups with equal gender composition [44,45], and more collaboration in same-gender groups than in mixed ones [44,46].

The time and effort spent on getting familiar with each other and coordinating may negatively affect the learning process and outcomes [43]. This argument finds support in research reporting collaborative learning with familiar partners (typically defined as related family members such as a spouse) being more effective than that with unfamiliar partners in enhancing cognitive performance [47] or in reducing the negative effects of collaboration [40,41]. Yet evidence exists that, even with familiar partners, collaborative learning does not generate more benefits than individualistic learning in improving older adults’ cognitive performance [42].

Ample evidence suggests that prior computer experience is a strong predictor for older adults’ computer task performance and learning outcomes [48-50]. Cody et al [28] in their study of older computer learners found that “the same [computer training] program was too challenging to some and insufficiently stimulating to others” (p 282). Some researchers [25] suggest forming homogeneous groups based on prior computer experience to ensure the success of computer training for older adults. This suggestion should be taken with caution, given that to date research on computer training for older adults has focused predominantly on individualistic learning while paying little attention to collaborative learning (an important exception is the Zandri and Charness study [51], which found promising signs for the superiority of collaborative learning over individualistic learning). While homogeneous groups based on prior computer experience might work better than heterogeneous groups in individualistic learning, this might not be the case for the collaborative learning condition.

### Research Questions and Hypotheses

The present study asked the following primary research question: what impact might the intervention have on older adults’ computer and Web knowledge, procedural skills, eHealth literacy efficacy, attitudes, and participation in their own health care?

Under this primary research question, 2 subresearch questions were asked: (1) what impact might the learning method (collaborative; individualistic) have on the learning outcomes?, and (2) what impact might group composition (based on gender, prior familiarity with peers, and prior computer experience) have on the learning outcomes?

The following hypotheses were tested: (1) hypothesis 1: computer/Web knowledge, skills, and eHealth literacy efficacy increase significantly from pre to post intervention (at the .05 level; same for all hypotheses), (2) hypothesis 2: collaborative learning is significantly more effective than individualistic learning in improving the learning outcomes, and (3) hypothesis 3: collaborative learning is significantly more effective than individualistic learning in heterogeneous group compositions, while individualistic learning is more effective than collaborative learning in homogeneous group compositions.

## Methods

### Design

We used a 2 × 2 mixed factorial design with learning method (collaborative; individualistic) as the between-participants variable and time of measurement (pre; post) as the within-participants variable.

### Research Sites

The Hyattsville and New Carrollton branch libraries of the Prince George’s County Memorial Library System served as the primary research sites for this study. The Library System is a publicly funded large, urban library system serving over 830,000 residents in Prince George’s County, Maryland, USA. It has been serving as the primary research site for the larger *e*HiLL research project since 2007 [4-6,24]. This Library System was selected as the key site for the *e*HiLL research project because it serves a large population of ethnic minorities, particularly African American/black people. According to the US Census Bureau, 66% of Prince George’s County residents are African American/black, much higher than the 30% overall rate of African American/black residents in Maryland or the 12% rate nationwide (http://www.census.gov). Partnering with this Library System ensures the reach of *e*HiLL interventions to individuals from this underserved minority group. The Hyattsville and New Carrollton branch libraries of the Library System provided free networked computers, space, and staff support to facilitate the implementation of this study. The geographic location of these branch libraries is convenient for potential research participants and the researchers. Both are within 10 miles of the University of Maryland and easily accessible by car or public transportation.

### Participants

Standard recruitment techniques were used to recruit participants. These included posting recruitment flyers in the branch libraries and other local organizations (eg, senior centers, community centers, and churches) and advertising in the Library System’s newsletter. The inclusion criterion was age 60 years and above, though on request and in cases where seats were available, we accommodated individuals a few years younger. A total of 146 older adults aged 56–91 years (mean 69.99, SD 8.12) participated in the present study during a 4-month period (February to May 2011). We randomly assigned 72 participants to the collaborative learning experimental condition and 74 to the individualistic learning condition. Table 1 summarizes participants’ basic characteristics, including demographics, health status, and prior computer experience.

**Table 1:** Participants’ basic characteristics

Variable		n	%
**Gender**			
	Female	96	69
	Male	44	31
**Highest level of education**			
	Less than high school graduate	15	11
	High school graduate/GED^a^	39	28
	Vocational training	11	8
	Some college/associate’s degree	33	23
	Bachelor’s degree	25	18
	Master’s degree or other postgraduate training	16	11
	Doctoral degree	2	1
**Ethnic group**			
	Asian	8	6
	African American/black	90	64
	White	30	21
	Other	13	4
**Household annual incomerange (US $)**			
	<20,000	38	28
	20,000–29,999	28	20
	30,000–39,999	14	10
	40,000–49,999	8	6
	50,000–59,999	9	6
	60,000–69,999	6	4
	70,000–99,999	4	3
	≥100,000	3	2
	Do not know for certain	12	9
	Do not wish to answer	18	13
**Health status**			
	Poor	7	5
	Fair	24	17
	Good	75	53
	Very good	25	18
	Excellent	11	8
**English as primary language**			
	Yes	125	88
	No	16	12

**Frequency of computer use**			
	Every day	12	9
	Every 2–3 days	27	20
	Once a week	18	13
	More than once a month	13	9
	Less than once a month	20	15
	Never	48	35

^a^ General equivalency diploma.

### Measures

Adapted from existing outcome measures of collaborative learning (that focus on younger learners in formal educational settings) and with necessary modifications based on the results of a prior *e*HiLL study [5], outcome measures (dependent variables) for the present study covered the following categories: knowledge gains, skill gains, efficacy, attitudes, and changes in participation in own health care. Learning preference, familiarity with peers, prior computer experience, and basic demographics were measured to serve as control variables. (Copies of the instrument are available on request to the author.)

#### Computer/Web Knowledge

This was measured by objective tests of knowledge about components of the computer and the Web. Participants were shown an image of a computer and a screenshot of the NIHSeniorHealth.gov website, and were instructed to write down names of the main components of each image (eg, keyboard, mouse, link, scroll bar). Computer knowledge and Web knowledge were each measured by 5 items; each item scored 1 point if answered correctly and 0 points if answered incorrectly with a scoring range of 0–5.

#### Computer/Web Skills

These were measured by procedural tests of the abilities to carry out specific computer and Web operations. Each participant had one computer to use during the testing. Participants were asked to perform a number of operations on their computers independently. Participants had up to 1 minute to complete each operation. Sample operations were to open a Web browser; go to the NIHSeniorHealth.gov Web site; increase text size; find information on the Falls and Older Adults health topic; and open a video. There were a total of 20 operations. Each operation scored 1 point if successfully completed and 0 points if unsuccessful, with a scoring range of 0–20.

#### eHealth Literacy Efficacy

This construct was measured by the eHealth literacy scale [52], which was built on the self-efficacy concept [53] to measure perceived skills at and comfort with using the Internet for health information and decision making. The main scale has 8 items. Each item is on a 1- to 5-point Likert scale with the following anchors: 1: strongly disagree; 2: disagree; 3: undecided; 4: agree; 5: strongly agree. Higher score indicates higher eHealth literacy efficacy. A sample item is “I know **how** to find helpful health resources on the Internet” (bold original). This scale has been used in multicultural samples and has shown excellent internal consistency reliability (scale alpha = .89–.97) with good test–retest reliability [23].

#### Attitude Toward the Class

This was measured by the following 5 items (items 3 and 4 were modified from Pace and Kuh [54]):

1. Overall, what would you say about the instructor’s teaching? (Anchors: 1: very poor; 2: poor; 3: fair; 4: good; 5: excellent)

2. Overall, was this computer class useful to you? (1: completely useless; 2: useless; 3: somewhat useful; 4: useful; 5: very useful)

3. How would you evaluate your entire experience in this computer class? (1: extremely dissatisfied; 2: dissatisfied; 3: neither satisfied nor dissatisfied; 4: satisfied; 5: extremely satisfied)

4. If you could start over again, would you go to the same computer class you are now attending? (1: definitely not; 2: probably not; 3: not sure; 4: yes; 5: definitely yes)

5. Would you want to recommend this training class to other people similar to your age? (1: definitely not; 2: probably not; 3: not sure; 4: yes; 5: definitely yes)

#### Changes in Participation in Own Health Care

This was measured by 12 items, including 6 items modified from a Kaiser survey study [16], 5 items modified from a Pew survey study [55], and an additional item added to supplement the Kaiser and Pew items. (These items are detailed in Table 6 in the Results section where the results are reported.)

#### Attitude Toward the Individualistic Versus Collaborative Learning Method

This was measured by the following item: “When I have to learn a new skill, I prefer to learn alone, rather than with others” [51]. It was scored on a 1- to 5-point Likert scale with the following anchors: 1: disagree strongly; 2: disagree; 3: undecided; 4: agree; and 5: agree strongly.

#### Prior Experience

Prior experience (familiarity) with peers was measured by the following item: “Are you related to or familiar with at least one person taking this same computer class? (eg, spouse, sibling, friend, acquaintance)”. Prior computer experience was measured by the frequency of computer use.

#### Basic Demographics

Age, gender, education, health status, race/ethnicity, income, and primary language were recorded.

Table 2 summarizes these measures and time(s) of measurement.

**Table 2:** Measures used in the present study and the time(s) of measurement

Variable	Pre	Post
Computer/Web knowledge	X	X
Computer/Web skills	X	X
eHealth literacy efficacy	X	X
Changes in health behavior/decision making		X
Attitude toward the class		X
Attitude toward the individualistic versus collaborative learning method	X	
Prior experience with peers	X	
Prior computer experience	X	
Basic demographics	X	

### Instructional Materials

This study used a set of instructional materials developed by the National Institute on Aging (NIA) of the NIH, “Helping Older Adults Search for Health Information Online: A Toolkit for Trainers” [56]. This freely available toolkit is designed to improve older adults’ ability to access and use NIH online health information resources (eg, the NIHSeniorHealth.gov website). By focusing on only NIH resources, this *e*HiLL intervention avoids potential problems associated with the quality of online health information [57,58]. The toolkit features detailed lesson plans, in-class interactive exercises, take-home practice exercises, and other supportive handouts (eg, a glossary of computer terms). This toolkit was chosen because, first, it contains key elements that, as addressed in the Introduction section above, facilitate older adults’ learning of computer technology [24-30]; and second, it was tested in prior *e*HiLL studies and proven to be effective [4,5,24].

The toolkit contains lesson plans (modules) designed to be used independently or in any combination. We used 4 modules in the present study to help older adults learn about (1) basic computer terms (1 module), (2) NIHSeniorHealth.gov (2 modules), a website designed to accommodate age-related changes in cognitive, physical, and sensory abilities [59], and (3) evaluating the quality of online health information (1 module). Together, these 4 selected modules provide a good coverage of the eHealth literacy skills as defined by Norman and Skinner [14]. Table 3 [56] outlines the lesson plans and goals of these 4 modules.

**Table 3:** Lesson plans and goals included in the National Institute on Aging (NIA) toolkit (extracted from the toolkit [56]).

Class session	Lesson goals
Session 1: Internet Basics (NIA Module #1)	1. Learn basic computer terms
	2. Practice using the mouse
	3. Learn basic Internet terms
	4. Learn how to get to a website
	5. Learn how to explore a website
	6. Learn how to use a search box
	7. Learn how to use a site map
Session 2: Introduction to NIHSeniorHealth (NIA Module #2)	1. Use the Home Page to find health topics on NIHSeniorHealth
	2. Use the Table of Contents of a health topic to find specific information
	3. Navigate through a health topic
	4. Enlarge, view, and close images
	5. Find answers to health questions of personal interest
Session 3: NIHSeniorHealth Quizzes and Videos (NIA Module #3)	1. Recall how to use the Home Page of the NIHSeniorHealth website
	2. Recall how to use the All Topics A–Z page to find health topics on the NIHSeniorHealth website
	3. Recall how to use the special features (optional)
	4. Learn how to take online quizzes
	5. Learn how to open, watch, and close a video
	6. Learn how to open, read, and close a video transcript
	7. Learn how to find answers to health questions of personal interest
Session 4: Evaluating Health Websites (NIA Module #9)	1. Reliable health information websites
	2. The sponsor of a health website
	3. The purpose of a health website
	4. The authors of the health information
	5. The reviewers of the heath information
	6. The most recent update of the health information
	7. The privacy policy of a health website
	8. Clues about the accuracy of a website’s health information
	9. The contact information for a health website

### Procedure

The general procedures of the present study are similar to those of prior *e*HiLL studies [4-6,24]. In the first session, participants first signed the consent form (approved by the Institutional Review Board of the University of Maryland). The pre intervention survey questionnaire was then administered, followed by the pre skill testing. The intervention began with the completion of the pre testing. At the end of the last session, the post intervention survey questionnaire was administered, followed by the post skill testing. Each class met twice a week, 2 hours each time between 9:00 and 11:00 am, for a total of 2 weeks, at a library site. Class size was small (no more than 8 participants per class). The instructors, trained Master of Library Science students, frequently provided immediate, positive, and useful feedback when needed. Each participant had one computer to work on during each session. The instructors emphasized hands-on practice and provided relevant handouts during each session. These procedural components were carefully designed based on the literature on older adults’ computer learning [24-30] and proven to be effective in prior *e*HiLL studies [4-6,24].

Compared with prior *e*HiLL studies [4-6,24], a unique procedural aspect of the present study was the use of both collaborative and individualistic learning methods. Building on prior work on computer class structure for older learners that focused primarily on individualistic learning [26] and the common strategies used to promote collaborative learning among younger adults in formal learning settings [33,34], and adapting those strategies to accommodate the special needs and preferences of older adult learners in the public library setting, we used several strategies during the sessions to promote individualistic or collaborative learning (Table 4).

**Table 4:** Class structure and strategies for the learning conditions

Activity/time	Individualistic learning	Collaborative learning
Housekeeping: 5 minutes	Welcome	Same as the individualistic condition
	Instructor self-introduction	
	Participants self-introduction	
	Practical information	
	How long the class session will last	
	Where the restrooms are	
	Environment check	
	Everyone has a computer	
	Everyone can see instructor	
	Everyone can hear	
Overview: 5 minutes	Goal statement	Same as the individualistic condition
	What the participants will know or be able to do after this class session	
	Agenda	
	What will happen during this session	
	Steps and procedure	
	What instructor will do and what participants will do	
Explanation of learning method: 1 minute	Explain explicitly that everyone in the class is expected to learn independently	Explain explicitly that the class is expected to learn together as a group
	Encourage participants to ask the instructor any questions that they might have	Encourage participants to share with and help peers with any questions that they might have (and explain that instructor will answer any remaining question)
Introduction to the specific topic of this class session: 5 minutes	Definitions, scope, background information	Same as the individualistic condition
Lecture and demonstration, step-by-step instruction (part 1): 20 minutes	Present material and demonstrate processes, following the instructions and examples used in the National Institute on Aging toolkit	Same as the individualistic condition
	Encourage questions	
	Get confirmation after each step is explained and demonstrated	
	Check frequently to make sure everyone is on the same page	
Brief reflection: 2 minutes	Pause briefly and instruct participants specifically to check their own notes and reflect independently	Pause briefly and instruct each participant specifically to compare notes with a peer sitting next to him or her and reflect together with peer
Continuation of lecture and demonstration: 20 minutes	Same as part 1 above	Same as the individualistic condition
Brief reflection: 2 minutes	Same as the first independent reflection session above	Same as the first collaborative reflection session above
Break: 5 minutes	Distribute handouts, which have in-class practice exercises and detailed, step-by-step instructions for completing the exercise	Same as the individualistic condition
Hands-on practice: 40 minutes	Participants perform the hands-on practice activity independently	Each participant pairs up with a peer to do the hands-on practice activity together
	Encourage participants to ask instructor questions about the specific steps of the exercise	Encourage participant to ask peers questions about the specific steps of the exercise
	Help to answer each participant’s questions	Participants discuss with peers and try to answer peers’ questions (if, after peer discussion and exploration, questions remain, then instructor will answer the remaining questions)
Practice vs group reflection: 10 minutes	Same as above (participants continue to engage in hands-on practice independently)	All participants (and instructor) sit in a circle to discuss, share, and reflect together
Closing: 5 minutes	Summarize content covered in this class session	Same as the individualistic condition
	Distribute handouts for take-home exercises, which have detailed, step-by-step instructions for completing the exercises	
	Point out opportunities for coming back to use the library’s computers to practice	
	Preview the topic of next class session	
	Thank participants for coming to this class session and remind them to come to the next class session	

### Data Analysis

The main statistical analyses conducted to test the hypotheses were various techniques of analysis of variance (ANOVA). These included multivariate repeated measures analyses, one-way ANOVA, and univariate analysis of covariance. Two-independent-samples tests (Mann-Whitney U) and chi-square tests were used to compare the collaborative versus individualistic samples.

## Results

### Comparing the Collaborative Versus Individualistic Experimental Groups

Two-independent-samples tests (Mann-Whitney U) found no significant difference in age (*P* = .13), education (*P* = .11), health (*P* = .85), income (*P* = .32), and computer use frequency (*P* = .06), and chi-square tests found no significant difference in gender (*P* = .66), ethnicity (*P* = .07), primary language (*P* = .81), and familiarity with peers (*P* = 1.00) among participants in the collaborative and individualistic experimental conditions. Chi-square test also found no significant difference in the retention rate of the 2 experimental conditions (*P* = .56); overall, a total of 108 participants completed both the pre and post testing, resulting in a 74% retention rate for this 2-week intervention. These results suggest the 2 experimental groups were comparable in these aspects.

### Comparing Participants Who Completed the Intervention Versus Those Who Did Not

Mann-Whitney U tests found no significant difference in age (*P* = .51), education (*P* = .41), health (*P* = .42), income (*P* = .78), and baseline computer knowledge (*P* = .80), Web knowledge (*P* = .81), skills (*P* = .70), and eHealth literacy efficacy (*P* = .12) between participants who completed both the pre and post testing and those who completed only the pre testing. Significant difference was found in computer use frequency, with participants who completed both the pre and post testing reported having *less* prior use of computers than those who completed only the pre testing (*P* = .007).

### Changes From Pre to Post Intervention

Multivariate repeated measures analyses found that, overall, computer knowledge, Web knowledge, procedural skills, and eHealth literacy efficacy improved significantly from pre to post intervention (*F*_4,90_ = 119.60, *P* < .001). Univariate repeated measures analyses revealed significant improvement from pre to post intervention on each of these 4 measures (*P* < .001 in all 4 cases; computer knowledge: *F*_1,93_ = 60.60; Web knowledge: *F*_1,93_ = 54.92; procedural skills: *F*_1,93_ = 264.40; and eHealth literacy efficacy: *F*_1,93_ = 229.31). Hypothesis 1 was strongly supported. Further, effect sizes (measured by Cohen's d) with regard to gains from pre to post intervention in computer and Web knowledge, skills, and eHealth literacy efficacy ranged from 0.88 to 2.25. The statistical power of these measures reached 1.00 even at the alpha = .01 level (Table 5).

**Table 5:** Effect sizes and statistical power

Variable	Cohen's d	Percentile standing	Statistical power (alpha **=** .01)
Computer knowledge	1.05	84	1.00
Web knowledge	0.88	80	1.00
Procedural skill	1.70	95	1.00
eHealth literacy efficacy	2.25	99	1.00

### Attitudes

Participants in both the collaborative and individualistic learning conditions had overwhelmingly positive attitudes toward the intervention. Across the 2 conditions, 99% (94/95) of participants felt the instructor’s teaching was good or excellent; 95% (90/95) of participants felt the intervention was useful or very useful; 98% (92/94) were satisfied or extremely satisfied with the entire experience participating in the intervention; 84% (79/94) said they would attend the same class if it started over; and 98% (93/95) would recommend the intervention to their age peers. Multivariate ANOVA found no significant difference between participants in the collaborative and individualistic conditions in all measures of attitude.

It is worth noting, though, that 1 attitudinal measure, the instructor’s teaching, *approached* a statistically significant difference between the 2 experimental conditions (*F*_1,104_ = 3.34; *P* = .07). While participants in both conditions had very positive assessment of the instructor’s teaching, participants in the individualistic learning condition had a slightly more positive attitude toward the instructor’s teaching than those in the collaborative learning condition (individualistic: mean 4.87, SD 0.35; collaborative: mean 4.72, SD 0.45).

### Changes in Participation in Own Health Care

Across the collaborative and individualistic learning conditions, a notable number of participants reported changes in various aspects of participation in their own health care (Table 6).

**Table 6:** Changes in participation in own health care as a result of the intervention

	Yes		No	
	n	%	n	%
Since you started taking this computer class, have you had a conversation with a friend or family member about the health information you found on NIHSeniorHealth?	55	59	39	42
Since you started taking this computer class, have you talked with a doctor or other health care provider about the information you found on NIHSeniorHealth?	9	10	84	90
Have you changed your behavior because of the health information you found on NIHSeniorHealth?	51	55	41	45
Have you made a decision about how to treat an illness or condition because of the information you found on NIHSeniorHealth?	56	61	36	39
Have you changed your health insurance plan because of the information you found on NIHSeniorHealth?	2	2	86	98
Have you changed your overall approach to maintaining your health or the health of someone you help take care of because of the information you found on NIHSeniorHealth?	58	64	33	36
Has the information you learned from NIHSeniorHealth led you to ask a doctor new questions or to get a second opinion from another doctor?	54	62	33	38
Has the information you found on NIHSeniorHealth changed the way you think about diet, exercise, or stress management?	69	76	22	24
Has the information you found on NIHSeniorHealth changed the way you cope with a chronic condition or manage pain?	51	57	38	43
Has the information you found on NIHSeniorHealth affected a decision about whether to see a doctor?	37	42	52	58
Have you changed the way you take medicine because of the information you found on NIHSeniorHealth?	32	36	58	64

One-way ANOVA found no significant difference in the total number of reported changes in participation in own health care between participants in the collaborative and individualistic learning conditions (*P* = .45).

### Collaborative Versus Individualistic Learning Method

Multivariate repeated measures analyses found a significant main effect of the learning method on computer knowledge, Web knowledge, procedural skills, and eHealth literacy (*F*_4,90_ = 4.56, *P* = .002). To examine on which specific outcome measure(s) the learning method showed a main effect, univariate repeated measures analyses were performed and revealed a significant main effect of the learning method on procedural skills (*F*_1,93_ = 7.81; *P* = .006) and eHealth literacy (*F*_1,93_ = 8.64; *P* = .004). Univariate analyses revealed no significant main effect of the learning method on either computer knowledge (*P* = .51) or Web knowledge (*P* = .47).

Interestingly, one-way ANOVA found a significant difference in pre intervention procedural skills (*F*_1,142_ = 7.17; *P* = .008) and eHealth literacy (*F*_1,140_ = 6.18; *P* = .01) between participants in the individualistic and collaborative learning groups. Pre intervention computer knowledge (*P* = .90) and Web knowledge (*P* = . 94) did not differ significantly between the 2 experimental groups. To examine whether the significant main effects of the learning method on procedural skills and eHealth literacy as revealed by the univariate analyses were due to significant differences in these variables at the baseline, univariate analysis of covariance was performed and, after controlling for pre intervention procedural skills and eHealth literacy, respectively, the significant effects of the learning method on these variables both disappeared (procedural skills: *P* = .36; eHealth literacy: *P* = .06). These findings suggest that, when controlling for these variables at the baseline, the learning method had no main effect on knowledge, skills, and eHealth literacy efficacy. Hypothesis 2 was not supported.

Multivariate repeated measures analyses found no significant interaction effect of the learning method (collaborative; individualistic) and time of measurement (pre; post): *P* = .73.

### Group Compositions

Groups in this study had 4 gender compositions (there was no male-only group): female-only (14 participants in this group composition), female majority (n = 96), equal number of female and male (n = 10), and male majority (n = 13). Multivariate repeated measures analyses found no significant 3- or 2-way interaction effect of group gender composition, learning method, and time of measurement (learning condition × gender composition: *P* = .40; time × gender composition: *P* = .74; time × learning condition × gender composition: *P* = .79). Further, group gender composition had no significant main effect on the outcome measures (*P* = .68).

Groups had 2 compositions based on familiarity with peers: familiar with at least one other person in the same session (n = 37) and not familiar with anyone in the same session (n = 106). Multivariate repeated measures analyses found no significant 3- or 2-way interaction effect of group familiarity composition, learning method, and time of measurement (learning condition × familiarity composition: *P* = .37; time × familiarity composition: *P* = .80; time × learning condition × familiarity composition: *P* = .88). Further, no significant main effect of group familiarity composition was found (*P* = .53).

Similar to 2 prior *e*HiLL studies [5,6], computer use frequency was used to categorize group composition based on prior computer experience. “Experienced computer users” were defined as individuals who use the computer every day or every 2–3 days a week, and “inexperienced computer users” were defined as individuals who use the computer less than every 2–3 days a week. Using this criterion, we coded each session into 1 of the following 5 groups: inexperienced user only (n = 36), inexperienced user majority (n = 88), equal number of experienced and inexperienced users (n = 4), experienced user majority (n = 10), and experienced user only (n = 1). These groups were further recoded into 2 groups: groups with mixed users (n = 102) and groups with either all inexperienced users or all experienced users (n = 37). Multivariate repeated measures analyses found no significant 3- or 2-way interaction effect of group composition based on prior computer experience, learning method, and time of measurement (learning condition × prior computer experience composition: *P* = .40; time × prior computer experience composition: *P* = .20; time × learning condition × prior computer experience composition: *P* = .53). Further, no significant main effect of group composition based on prior computer experience was found (*P* = .54). Hypothesis 3 was not supported.

### Learning Preferences

To examine the potential impact of attitude toward the individualistic versus collaborative learning method (learning preference), participants were recoded into a “matched” group, in which participants’ learning preferences matched their collaborative versus individualistic group assignments, and a “no-match” group, where the preference and the group assignment did not match. Multivariate repeated measures analyses found neither an interaction effect among learning preference matching, learning method, and time of measurement (*P* = .18) nor a significant main effect of the learning preference matching factor (*P* = .41).

## Discussion

Older adults are in great need of health literacy interventions, given that their needs for health information and services are typically high [17,60-62] and yet their health literacy levels are low [8]. Due to age-related changes in social environments and individual abilities [63], interventions that target younger age groups are unlikely to reach or have similar impact on older adults. Further complicating the situation is that the requirement for health literacy skills is a moving target, particularly in the context of eHealth becoming increasingly prominent in contemporary society [9,10]. As Norman [23] correctly points out, as technology changes, so do the requirements for health literacy skills.

### Impact of the Overall Intervention

This study aimed to generate new scientific knowledge about effective eHealth literacy interventions for the older population. The primary research question was “What impact might the intervention have on the learning outcomes?” The analyses revealed that computer knowledge, Web knowledge, procedural skills, and eHealth literacy efficacy all improved significantly from pre to post intervention (*P* < .001 in all cases). The effect sizes of these improvements ranged from 0.88 to 2.25, suggesting that the magnitude of these improvements was large [64]. What these effect sizes mean is that, for instance, with respect to improvements in eHealth literacy efficacy, an effect size of 2.25 meant a learner increased from the 50th percentile on the pre test to the 99th percentile on the post test on this measure. These results strongly support the magnitude of the effects of the intervention. Further adding to the strength of these positive results is that the statistical power of these measures was strong: it reached 1.00 even at the alpha = .01 level in all cases.

These findings are even more impressive when interpreted in the context of the literature showing that “effect sizes of 0.8 are rare for any [learning] intervention and require truly impressive gains” ([31] p 224). Also, as summarized in several meta-analyses, the effect sizes of prior collaborative learning interventions (that focused on younger learners in formal educational settings) ranged from 0.29 to 0.70 [33,65,66]. In the present study, the effect size of the intervention on all knowledge, skill, and efficacy measures was greater than 0.8, suggesting the intervention has indeed resulted in “truly impressive gains.” These results provide strong support that the intervention, regardless of the specific learning method used, was effective in improving older adults’ eHealth literacy.

Across the 2 experimental conditions, participants had overwhelmingly positive views of the intervention. Notable percentages of participants also reported changes in various aspects of participation in their own health care. These findings suggest the health information these participants obtained from the intervention had affected their health behavior and decision making, which is a key component of eHealth literacy [14]. These findings further suggest that the intervention, regardless of the specific learning method used, was effective in improving older adults’ eHealth literacy. These findings are particularly meaningful in the context of the contemporary health care system increasingly promoting shared medical decision making, where patients are expected to participate more in their own health care [60,67-71].

An important reason for the effectiveness of the intervention tested in this study was that it fully incorporated the key elements of successful computer training for older adults [24-30], as outlined in the Introduction section above. These key elements were proved effective in prior *e*HiLL studies [4-6,24]. A unique aspect of this study was to compare the effectiveness of the collaborative and individualistic learning methods built into the intervention. The analyses yielded interesting findings, as discussed in the next subsection.

### Collaborative Versus Individualistic Learning Method

The analyses found neither an interaction effect of the learning method and time of measurement nor a main effect of the learning method on any of the outcome measures, suggesting the collaborative and individualistic learning methods did not differ in their relative effects. This finding deserves careful consideration. As reviewed above, the superiority of collaborative learning over individualistic learning, as is well documented in the literature, is based on studies of younger learners in formal educational settings [33,34]. It is possible that collaborative learning may simply not work as well for the older population in informal settings. If this is the case, then this study contributes to the literature by identifying some key limits of the social interdependency theory.

In particular, the sample of this study consisted of primarily inexperienced computer users: less than 9% of participants used computers every day, while 35% of participants had never used a computer before (participants who used computers less often than every 2–3 days made up 72% of the study sample). Engaging in collaborative learning might have been too challenging for most participants who had to focus on their own activities with little attention to spare to interact with and help others, which is in line with the findings of prior research [25,28]. Thus, the previously widely reported advantages of collaborative learning over individualistic learning may not be easily realized among individuals who have limited prior computer experience.

Another way to look at this matter, however, is that perhaps different collaborative learning strategies could be used to better promote collaboration among inexperienced computer users. The collaborative learning strategies used in this study were carefully developed based on prior research on younger adult learning in formal educational settings [33,34] and modified to accommodate the older population in informal settings (see Table 4). In reflection, however, there might not have been sufficient consideration for the largely inexperienced user composition of the study sample.

For logistical reasons, multiple instructors were hired on an hourly basis to provide the instructions. It is likely that individual differences in these instructors (eg, teaching style, commitment, personality) may have affected the learning outcomes. One indication of this possibility is that the measure of the instructor’s teaching *approached* a statistically significant difference between the 2 experimental conditions (*P* = .07). Participants in the individualistic learning condition expressed more positive views of the instructor’s teaching than those in the collaborative learning condition. It is possible that this factor might have helped at least partially offset any hypothesized advantage of collaborative learning over individualistic learning.

The ceiling effect might have also affected the results: due to the positive impact of either version of the intervention, it may have been difficult to differentiate between the relative effects of the collaborative versus individualistic versions of the intervention. A possible solution for future research is to make the knowledge and skill tests more challenging so that the measures can be more sensitive to potential differences in learning outcomes.

### Group Composition

As reviewed above, the literature suggests that group composition factors (based on gender, prior familiarity with peers, and prior computer experience) may affect the learning outcome. This study, however, found neither an interaction effect of any of these group composition factors with the collaborative versus individualistic learning method nor a main effect of any of these group composition factors. These findings are in line with those of 2 earlier *e*HiLL studies [5,6]. Replicating the same findings in these 3 independent studies, which differed in multiple ways (eg, intervention duration, instructional materials, procedures, and participants), lends some support to the generalizability of these findings. Note, though, that the study samples of these 3 independent *e*HiLL studies were similar: in each study the majority of the participants were women, unfamiliar with their study peers, and inexperienced computer users. While these findings might be generalized to populations with similar characteristics, they may not be so to populations with different characteristics. Also, as discussed above, if there were insufficient strategies to fully promote collaborative learning, then the potential effect of group composition might have also been affected.

Participants who completed both the pre and post testing reported *less* prior use of computers than those who completed only the pre testing (*P* < .01), suggesting that participants who used computers more often were more likely to drop out. One possible reason is that the intervention, by design, started from basic computer terms and increased in complexity gradually (see Table 3). Yet this might not have been made clear to the participants and some of them, after the first session, might have gotten the impression that the class was too “basic” for them and thus left. In future research, it will be necessary to fully communicate to participants, during the very first session, all the topics that will be covered in the remaining sessions.

### Practical Implications

As in the earlier *e*HiLL interventions [4-6,24], the intervention tested in the present study also involved productive partnerships among local public libraries, a library and information science academic program at a state university, and the NIH. These local, state, and federal organizations bring complementary resources to the project and, in doing so, help each organization to achieve its mission [4]. The local public libraries provide the facility and staff support for the project, helping the libraries better serve socially, economically, and technologically underserved library patrons. The library and information science academic program provides the human resources through well-trained and dedicated faculty and graduate students and, in doing so, better achieves its research and educational missions. The NIH provides reliable online health information resources, and its involvement in this study helps promote the use of these resources. Tapping into these well-established public infrastructures ensures the intervention’s capacity for scaling up (eg, it can be easily rolled out to other communities across the country to improve older adults’ eHealth literacy).

### Limitations and Future Directions

This study has some limitations. First, the sample was a convenience one, consisting of mostly African American/black people (64%), women (69%), and inexperienced computer users (72%). The findings may not be representative of the older population as a whole and should not be generalized without caution. Future research will benefit from examining the issues in a representative sample.

Second, potential confounding factors might have affected the relative effects of collaborative versus individualistic learning. These include insufficient consideration for the inexperienced user composition of the sample, instructor differences, and the ceiling effect. It will be necessary in future research to test strategies that can more fully promote collaboration among participants who have limited computer experience, control for instructor differences, and use more sensitive measurements to eliminate a potential ceiling effect.

Third, eHealth literacy skills may involve different levels of skills, with some skills being relatively easier to obtain and others requiring more effort. In future research, it will be necessary to develop more refined measures to assess changes in skills on different levels (eg, skills in not only finding a particular health topic on the NIHSeniorHealth.gov site but also determining the quality of information on any health website).

Fourth, this experimental study did not have a qualitative component. Future research can include qualitative data collection and analysis, which may generate additional insights into the learning process and the relative effects of collaborative versus individualistic learning.

Fifth, this study did not include measures of participants’ potential practice of the skills outside of the intervention (eg, at home or other locations). Future research may include these measures to determine whether and how outside practice might affect the effects of the intervention.

Finally, changes in participation in own health care were measured in this study by self-report and at only one time point (post intervention) with no follow-up beyond the intervention period. In future research, it will be necessary to add more objective measures (eg, physicians’ reports) and measure this variable over time at multiple time points.

### Conclusions

The findings of this experimental study contribute to the literatures on adult learning, social interdependence theory, and health literacy. This study used both objective and self-report measures. The findings from both types of measures are consistent and, together, provide strong evidence that the eHealth literacy intervention tested in this study, regardless of the specific learning method used, significantly improved knowledge, skills, and efficacy. Participants also had overwhelmingly positive attitudes toward the intervention. Participants reported changes in participation in their own health care as a result of the intervention, further supporting the effectiveness of the intervention on improving older adults’ eHealth literacy. Collaborative learning did not differ from individualistic learning in affecting the learning outcomes, suggesting that the previously widely reported advantages of collaborative learning over individualistic learning may not be easily applied to the older population in informal settings, though several confounding factors might have contributed to this finding. Further research is necessary before a more firm conclusion can be drawn. Finally, group composition based on gender, familiarity with peers, and prior computer experience demonstrated no significant interaction or main effect on the learning outcomes.

The study addressed an important social problem: the health “illiteracy” problem among older adults, particularly those who have low incomes, limited education, limited prior computer/Internet experience, and/or belong to ethnic minority groups. The findings of this study contribute to scientific knowledge by advancing theory in older adult learning, particularly the generalizability and application of the collaborative versus individualistic learning method to the older population in an informal setting, and the use of these learning methods as an effective eHealth literacy intervention. By focusing the content of learning on eHealth literacy knowledge and skills, this study broadens current understanding of the health literacy concept and interventions to address the increasing importance of technology in health care. By developing and testing the effectiveness of an eHealth literacy intervention, this study shapes this newly emerging component of health literacy (ie, eHealth literacy) that has increasing significance in contemporary health care.

This study broadens current paradigms in health literacy by using concepts and approaches novel to the field of health literacy. First, while health literacy has been promoted as a lifelong learning process [22], little attention has been paid to examining the relative benefits of different instructional methods (eg, individualistic versus collaborative learning) on older adults’ learning of health literacy knowledge and skills. Second, while some prior interventions have involved the use of computers, their primary approach is presenting medical materials on a specific topic through a specially designed localized computer-based system [21]. While such an approach has its advantages (eg, targeting a very specific problem), it also has limitations: it requires extensive resources (to develop and update), and the knowledge and skills learned through this approach are often difficult to generalize to other areas or computer systems. In contrast, in the present study, we used the high-quality Internet health information resources maintained and updated by the NIH that focus on a broad range of medical knowledge, and provide training to improve general knowledge about and skills in finding information on any health topic that might be of interest to an individual—and from a common computer system. Thus, this *e*HiLL intervention is cost effective and easily transferrable.

## Abbreviations

ANOVA: analysis of variance
eHiLL: Electronic Health Information for Lifelong Learners
